# Maternal microbiota and gestational diabetes: impact on infant health

**DOI:** 10.1186/s12967-023-04230-3

**Published:** 2023-06-06

**Authors:** Parul Singh, Duaa Ahmed Idris Elhaj, Ibrahim Ibrahim, Hala Abdullahi, Souhaila Al Khodor

**Affiliations:** 1 College of Health & Life Sciences, Hamad Bin Khalifa University, Qatar Foundation, Doha, Qatar; 2 Research Department, Sidra Medicine, Doha, Qatar; 3 Women’s Department, Sidra Medicine, Weill Cornell Medical College-Qatar, Doha, Qatar

**Keywords:** Microbiome, GDM, Neonatal health, 16S rRNA

## Abstract

Gestational diabetes mellitus (GDM) is a common complication of pregnancy that has been associated with an increased risk of obesity and diabetes in the offspring. Pregnancy is accompanied by tightly regulated changes in the endocrine, metabolic, immune, and microbial systems, and deviations from these changes can alter the mother’s metabolism resulting in adverse pregnancy outcomes and a negative impact on the health of her infant. Maternal microbiomes are significant drivers of mother and child health outcomes, and many microbial metabolites are likely to influence the host health. This review discusses the current understanding of how the microbiota and microbial metabolites may contribute to the development of GDM and how GDM-associated changes in the maternal microbiome can affect infant’s health. We also describe microbiota-based interventions that aim to improve metabolic health and outline future directions for precision medicine research in this emerging field.

## Introduction

Pregnancy is a complex process that is influenced by a variety of interconnected molecular and cellular mechanisms [1]. During pregnancy, there are many physiological changes that occur, including hormonal, immunological, microbial, and metabolic changes, which are all tightly regulated to help maintain homeostasis and ensure the delivery of a healthy infant [1, 2]. However, if these physiological changes are disrupted, various pregnancy-related complications can occur leading to negative consequences for both the mother and her baby [3]. There has been increasing interest in studying the role of microbiota in reproductive health and associated changes during pregnancy and newborn life.

Indigenous microbial communities, also known as the microbiota, form intricate ecosystems that are uniquely adapted to the constantly fluctuating physiology of their hosts [4]. Three-quarters of an individual’s microbiome can be traced back to their mother, with the infant being exposed to vaginal microbes as they pass through the birth canal [5]. Additionally, maternal oral [6], fecal [7, 8], skin [9] and placental [10] microbiota can also contribute to the seeding and colonization of the infant microbiome. Breastmilk plays a role in the maturation and nourishment of the infant microbiome after birth [11]. Microbiome imbalance (also known as dysbiosis) may affect the mother's metabolic profile, contribute to pregnancy complications, and impact neonatal health [12].

Gestational diabetes mellitus (GDM) is defined as an abnormal glucose intolerance during pregnancy [13]. It has been linked to numerous adverse maternal and neonatal outcomes, such as cesarean section delivery, preeclampsia, large birth weight, shoulder dystocia, and hypoglycemia in newborns [13]. The prevalence of GDM is increasing and it affects a significant percentage of pregnancies [13]. Research has shown that the maternal microbiome may be altered in GDM pregnancies in multiple body sites, including the mouth, vagina, and gut [14]. However, the impact of the maternal microbiome changes on the composition and development of the infant's gut microbiome is still being explored. Differences in the composition of breast milk was shown to contribute to the alterations in gut microbiome composition in breastfed infants of obese mothers [15]. Proper seeding and maturation of infant gut microbiota is necessary for the development of various essential biological systems. The gut microbiome, also known as "the second brain," is composed of microbes that play a crucial role in various physiological processes, including digestion, immunity, neurological signaling, hormonal regulation, drug and toxin metabolism, and the production of metabolites that influence host physiology [16]. Microbiota ferment non-digestible fibers to produce microbial metabolites, such as short chain fatty acids (SCFAs) such as acetate, butyrate and propionate, that play a crucial role in maintaining the integrity of the intestinal barrier, preventing the leakage of pathogenic bacteria and toxic bacterial byproducts, such as lipopolysaccharide, into the bloodstream [17].

It is important to understand the relationship between the maternal microbiome and the health of the infant. This review will discuss the existing and updated knowledge about the physiological changes associated with normal pregnancy and compare them to those with pregnancy related complications such as GDM. We will further explore the relationship between the maternal microbiome, microbiota-derived metabolites, and GDM, as well as their impact on the development of the neonatal microbiome during the perinatal period and early life. We will also summarize various tools for modulating or engineering the microbiome in order to promote the development of a healthy, well-balanced, resilient microbiome that can protect the host from disease throughout their life.

### Pregnancy-related maternal changes

**Fig. 1:**
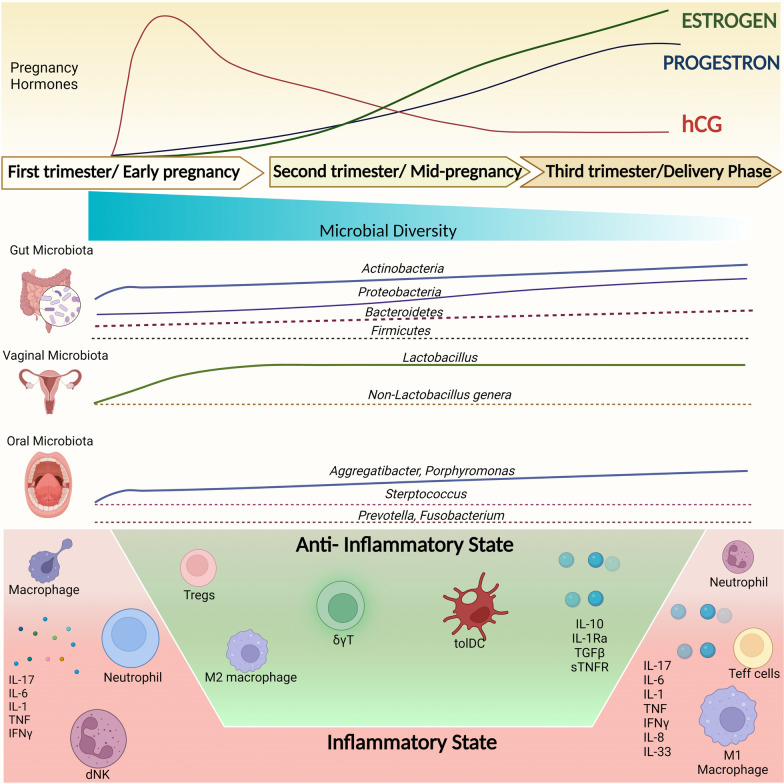
Maternal hormonal, microbial and immunological adaptations over the course of pregnancy. Created with Biorender.com

Physiological changes that occur in the mother's body during pregnancy are necessary to support the growing fetus and prepare the body for childbirth. Some examples of maternal adaptations during pregnancy include changes in hormone levels, weight gain, immune and microbial modulation [12] (Fig. 1).

### Hormonal adaptation during pregnancy and GDM

During pregnancy, there are several hormonal changes that occur in the mother's body to prepare the mother for delivery and to support her growing fetus. Human chorionic gonadotropin (hCG) is a hormone that can be detected in the urine as early as 7–9 days after fertilization and is used as an indicator of pregnancy [18]. As hCG levels rise, blood flow to the kidneys and pelvic region increases, which speeds up the kidneys' ability to eliminate waste from the body [18]. In addition, hCG regulates the levels progesterone and estrogen [18]. Progesterone levels must remain high during pregnancy and gradually increase until birth [19]. Progesterone has many important functions in the placentation and implantation [19] and later in pregnancy to support fetal development and prepare the muscles of the pelvic wall for contraction during labor [19]. Similarly, estrogen is made and released by the corpus luteum of the ovaries and later by the fetal-placental unit [20]. Estrogen levels gradually increase in pregnancy until delivery and are essential for maintaining and regulating the production of other pregnancy hormones, contributing to the development of fetal organs, stimulating the growth and function of the placenta, and preparing the mother for lactation [20]. These hormonal steroids influence many aspects of maternal physiology that are beneficial for both pregnancy and postpartum support for the infant [20]. Pregnancy is associated with a natural increase in insulin resistance, some studies have suggested that high levels of hCG and low levels of estrogen may be associated with the development of GDM [21–24], while others have found no significant changes in placental hormones in women with GDM [24]. The role of these hormones in the onset or progression of GDM remains largely unknown. Several other placenta-derived hormones with pleiotropic effects are also produced during pregnancy but are beyond the scope of this review.

### Pregnancy-associated immune adaptation and GDM

A pregnant woman's immune system undergoes significant changes in order to accommodate the developing fetus (Fig. 1). In a delicate balancing act, the maternal immune system supports fetal allograft tolerance while conserving innate and adaptive immune systems for defense against microbes [25]. A healthy pregnancy is characterized by a well-controlled mild inflammatory state, while the adaptive immune response is more active during implantation, birth, and intrauterine infections, the innate immune response continuously monitors the maternal–fetal interface for foreign antigens that could harm the pregnancy [25]. The process of implantation involves the production of pro-inflammatory cytokines such as IFNγ, IL-1, TNF, IL-6, IL-17 and the IL-6 family cytokine leukemia inhibitory factor (LIF) by immune cells such as natural killer (NK) cells, dendritic cells (DC), macrophages, neutrophils, and ILC3s [26, 27]. Mid-gestation is predominated by M2 macrophages, which produce anti-inflammatory cytokines such as IL-10 and TGF-β, dominate [28]. This is a time of rapid fetal growth and protection against preterm contractions [28]. There are fewer NK cells present in the second trimester, and their numbers continue to decrease until they are no longer present [28]. As a result, pregnancy enters a state of tolerance, and regulatory proteins control the function of tolerogenic dendritic cells, expand Treg cells, calibrate the function of NK cells, and downregulate effector T cells [29]. Towards the end of pregnancy, cytokines produced by the innate immune cells induce the pro-inflammatory process of parturition. Neutrophils and macrophages infiltrate the decidua and the chorioamniotic membranes during term labor and secrete matrix metalloproteinases, IL-1, IL-6, TNF and nitric oxide (Fig. 1) [30].

In women with GDM, studies have shown that macrophage infiltration is significantly increased in the omental visceral adipose tissue compared to healthy pregnancies, which significantly correlates with maternal insulin resistance [31]. This increased infiltration of macrophages is correlated with maternal insulin resistance [31]. In addition, overweight women with GDM have an increased cytotoxic capacity, as indicated by higher levels of cytotoxic NK cells, and increased production of the cytokines IFN-γ and TNF-α, while TGF-β expression is reduced, compared to overweight women with uncomplicated pregnancies [32]. However, studies have found no significant change in the number of peripheral DCs in pregnancies with GDM compared to uncomplicated pregnancies [33]. Other major immune/metabolic/hormonal changes in GDM pregnancies compared to normal have been elucidated inFig. 2. Further research into the immune pathways involved in GDM may help to further elucidate its pathophysiology.

**Fig. 2:**
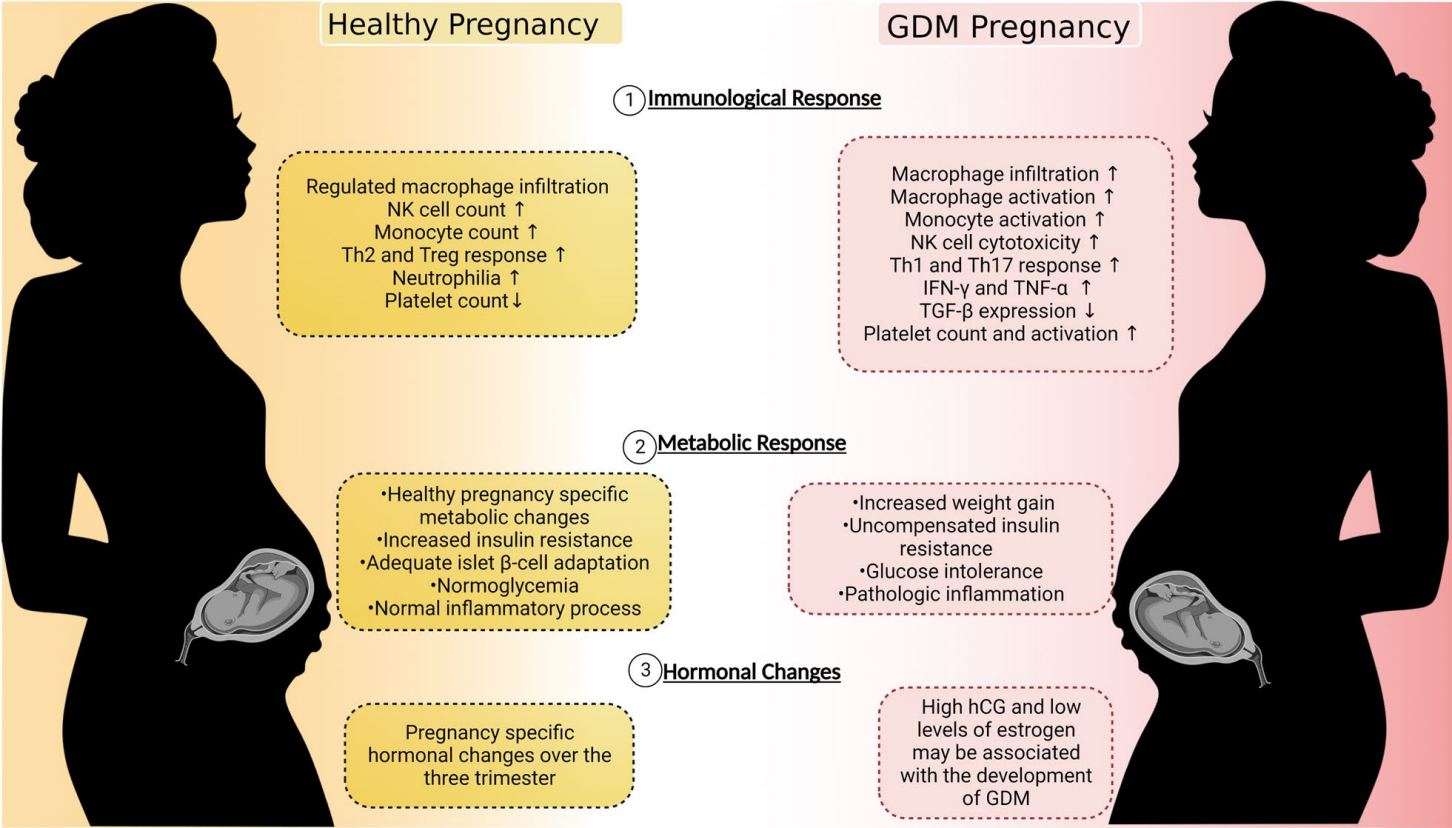
A portrayal of immune/metabolic and hormonal changes in GDM pregnancy compared to normal pregnancy. Created with Biorender.com. ↑, increased; ↓, decreased; GDM, gestational diabetes mellitus; NK, natural killer; Th2, T-helper 2 cell; Th1, T-helper 1 cell; Th17, T-helper 17 cell; Treg, regulatory T cell

### Metabolic changes in pregnancy and GDM

During pregnancy, the body undergoes metabolic changes similar to those seen in metabolic syndrome, including weight gain, insulin resistance, glucose intolerance, and low grade inflammation [34]. Pregnancy involves a complex regulation of the endocrine and metabolic systems. The ‘‘maternal anabolic phase’’ of the first trimester is marked by an increase in the mother's energy reserves, primarily in the form of lipids, which are stored to provide the substrates needed for advanced gestation and breastfeeding. ‘‘Maternal catabolic phase’’ or ‘‘fetal anabolic phase’’ is the term used to describe the second trimester of pregnancy, because it is aimed at fetal growth, with a significant reduction of insulin sensitivity and an increase in maternal concentrations of glucose and free fatty acids [35, 36].

The high levels of estrogens, progesterone, and insulin during the first and second trimesters of pregnancy also promote lipid deposition [37]. This is facilitated by increased expression of lipoprotein lipase and synthesis of fatty acids, which allow cells to more easily absorb circulating triacylglycerols [38]. Blood levels of fatty acids, triacylglycerols, cholesterol, and phospholipids continue to increase throughout the third trimester [39]. However, due to a shift towards a catabolic state, the accumulation of fat mass slows during the third trimester with increased lipolysis of adipose tissue. Lipids become the primary energy source for the mother during this time, while glucose and amino acids become retained for the fetus [37]. During the third trimester of pregnancy, there is a progressive reduction in blood glucose levels (from 75 to 65 mg/dL) [40]. Despite this drop in blood sugar and a 3.0 to 3.5-fold increase in fasting insulin, the mother's liver produces more glucose to meet the carbohydrate needs of the growing fetus and placenta [41, 42]. However, women who are obese or have a history GDM often have pre-existing insulin resistance that worsens as pregnancy progresses [43]. Compared to women with normal glucose tolerance, women with GDM have significantly higher levels of triglycerides, LDL cholesterol [44–46]. Metabolomics has been recently acknowledged as a helpful tool for the identification of metabolic disruptions associated with cardiovascular and metabolic diseases, including GDM [47].GDM metabolomics studies focused mainly on metabolites that are derived from amino acids, carbohydrates, lipid metabolites, purines, uric acid and bile acid, in addition to its related metabolic pathways [48–52]. These studies showed differential blood levels of very low density lipoprotein (VLDL), high-density lipoprotein (HDL), lysophosphatidylcholine (LPC), alanine, glutamate, isoleucine, phenylalanine, tyrosine, C14:1(trans-9), cysteine, and Proline which was observed as early as first trimester [48–51, 53] indicating that differential metabolites levels can act as early diagnostic biomarker for GDM.

### Microbiome adaptation during pregnancy and GDM

The significance of the maternal microbiota in pregnancy has recently come to light due to the discovery of significant changes in the composition of the microbiome during pregnancy [34]. The effect of alterations in the maternal microbiota at different bodily sites (such as the gut, vagina, mouth, etc.) on pregnancy complications has not been adequately studied, despite the long-standing knowledge of pregnancy-related hormonal, metabolic, and immunological changes [54, 55]. With a focus on GDM, we investigate in this research if there may be a link between the maternal microbiome and pregnancy complications. During pregnancy, the maternal microbiome undergoes significant changes, as depicted in Fig. 1. These changes entail an increase in the microbial diversity and a significant shift in the taxonomic composition of the microbial community from the beginning of pregnancy until the third trimester [2, 56–59].

The gut microbiome of a pregnant woman undergoes changes that result in an increased abundance of certain microbial communities, such as Actinobacteria and Proteobacteria, but a decrease in overall alpha diversity [2]. Overall, the gut microbiome composition during the first trimester, is shown to be similar to the gut microbiome of the healthy non-pregnant women which then vary gradually through the second trimester [2, 34]. In contrast, the third-trimester gut microbiota resembles that of individuals with metabolic syndrome or obesity [2]. GDM associated changes in the composition of the gut microbiota have been observed in the first [60, 61], second [61], third trimesters [62, 63], and postpartum period [62]. During the first and second trimesters, GDM is associated with a lower microbial diversity in the gut, as well as decreased relative abundance of *Coprococcus* and *Streptococcus *[61]. These bacteria produce various metabolites such as butyrate and lactate that are necessary for maintaining a healthy gut and immune regulation during pregnancy [64–66]. Other studies have found that GDM is associated with an increased abundance of Actinobacteria at the phylum level and *Collinsella*, *Rothia*, and *Desulfovibrio* at the genus level, and some of these differences in the microbiome persisted for up to eight months after delivery [62]. Certain opportunistic pathogenic bacteria, such as *Bacteroides caccae*, *Bacteroides massiliensis*, and *Bacteroides vulgatus*, are higher in abundance in individuals with GDM and T2D suggesting that these bacteria may play a role in the development of hyperglycemia associated with these conditions [67, 68]. Several studies have shown correlations between the relative abundance of certain gut bacteria and the carbohydrate metabolism. For example, *Blautia* and *Eubacterium hallii* have been positively correlated with fasting blood glucose, while the relative abundance of *Faecalibacterium* was shown to be negatively correlated [59, 69] These species may serve as biomarkers for GDM, as their abundance is not affected by dietary changes. High blood glucose levels have also been linked to low levels of the *Faecalibacterium/Fusobacterium* ratio in the gut [14].

Changes in the vaginal and oral microbiome have also been noted during pregnancy [70, 71]. Several studies have indicated that microbial dysbiosis may be linked to various pregnancy complications [72, 73]. For instance, changes in the oral microbiota, such as an increase in *Porphyromonas gingivalis*, may lead to increased risk of infections and production of pro-inflammatory cytokines, which has been proposed as a contributing factor to pregnancy complications such as preterm birth (PTB) [58, 74, 75]. Several studies showed an association between dysbiotic vaginal microbiota during pregnancy and negative pregnancy outcomes such as GDM, pregnancy loss, and low birth weight [2, 70, 76–79]. *Lactobacillus* species are typically the most dominant bacteria present in the vagina during normal pregnancy, as they can produce lactic acid that helps maintain a low pH and prevent the growth of pathogenic bacteria [80]. Some studies have shown that higher levels of inflammatory cytokines may be associated with an increased presence of potential pathogenic bacteria [75, 80]. Those include *Megasphaera* and *Eggertella* species that have been linked to vaginal dysbiosis and pregnancy complications, suggesting a possible role in GDM [61, 81]. In addition, higher blood glucose levels have been found to be positively correlated with higher ratios of *Prevotella/ Aerococcus* in the vagina [14, 82]. *Prevotella* has been identified as a biomarker for preterm delivery [83–85], but further research is needed to understand its role in GDM.

Table 1 summarizes the major studies that have used 16S rRNA gene sequencing to compare the microbiome of women with and without GDM.

**Table 1:** Comparison of the microbiome of women with and without GDM

Study/PMID	Location	Sample details	Subject details/time of sampling	Sequencing methodology	Main findings (Microbiota signatures in GDM) compared to controls
[2]	Finland	Stool samples	15 GDM and 76 non-GDM (1st trimester, 3rd trimester, 1 month postpartum)	16S rRNA gene sequencing (V1–V2 region)	Depleted microbial richness at T1(Trimester 1)
[59]	Italy	Stool samples	41 GDM (within patient comparison at enrolment (24–28 weeks) and at 38 weeks of gestation	16S rRNA gene sequencing ( V3–V4 region)	*At study end compared to enrollment:* *Bacteroides, Collinsella and Rikenellaceae* *Blautia, Butyricicoccus, Clostridium, Coprococcus, Dorea, Faecalibacterium, L − Ruminococcus (Ruminococcus genus assigned to Lachnospiraceae family), and Lachnospiraceae*
[62]	Denmark	Stool samples	50 GDM and 157 non-GDM (3rd trimester, 8 months postpartum)	16S rRNA gene sequencing ( V1–V2 region)	Differentially abundant OTUs in Third trimester of pregnancy: Actinobacteria*, Collinsella, Rothia, Desulfovibrio, Blautia, Faecalibacterium* Postpartum: Actinobacteria, *Collinsella, Olsenella, Clostridium, Faecalibacterium, Bacteroides, Veillonella, Bavariicoccus, Clostridium *sensu stricto*, Clostridiaceae_1, Hafnia, Howardella, Dehalobacter*
[86]	Germany	Stool samples	42 post-GDM and 35 control (3–16 months post-partum)	16S rRNA gene sequencing (V4 region)	*Prevotellaceae* *Firmicutes*
[63]	China	Stool samples	Pregnant women with GDM (n = 30) and normal controls(n = 31) in third trimester	16S rRNA gene sequencing (V3–V4)	*Gammaproteobacteria, Hemophilus*
[87]	Denmark	Saliva samples	Pregnant women with GDM (*n* = 50) and normal glucose regulation (*n* = 160) in third trimester and 9 months postpartum	16S rRNA gene sequencing (V3-V4)	Third trimester of pregnancy *Actinobacillus paraheamolyticus* (OTU_196)*Neisseria* (OTU_387) Nine months after pregnancy *Bacteriodales* (OTU_142), *Treponema* (OTU_242) *Leptotrichia* (OTU_37), *Streptococcus* (OTU_183), *Neisseria* (OTU_387), unclassified *Bacteria* (OTU_76), *Weeksellaceae* (OTU_29) and *Atopobium* (OTU_382)
[63]	China	Saliva samples	Pregnant women with GDM (n = 30) and normal controls(n = 31) in third trimester	16S rRNA gene sequencing (V3–V4)	alpha-diversity, *Fusobacteria*, *Leptotrichia* *Selenomonas*, *Bifidobacterium*
[14]	China	Saliva samples	346 pregnant women (42.98% GDM +), 175 oral samples collected	16S rRNA gene sequencing (V3–V4)	*Proteobacteria* *Firmicutes*
[88]	China	Vaginal samples	25 pregnant women with GDM (n = 15) and normal controls (n = 10) in second and third trimester	16S rRNA gene sequencing (V4–V5)	*Lactobacillus jensenii, Lactobacillus listeri, Lactobacillus amylovorus, Lactobacillus fructivorans* *Lactobacillus salivarius*
[75]	Brazil	Vaginal samples	68 pregnant women with GDM (n = 26) and normal controls (n = 42) in third trimester	16S rRNA gene sequencing (V4)	*Bacteroides, Veillonella, Klebsiella, Escherichia-Shigella, Enterococcus, and Enterobacter*

Decrease in relative abundance

Increase in relative abundance

Studies have also looked at the interactions between the microbiota and other physiological systems that play a critical role in pregnancy. For example, it was shown that higher levels of progesterone during the third trimester is correlated with an increase in the relative abundance of *Bifidobacterium* in pregnant mice [89] highlighting the possible link between *Bifidobacteria* and the dysregulated hormonal balance. Other studies have found that the presence of certain genera, including *Marvinbryantia*, *Acetivibrio*, *Gemminer*, *Bifidobacterium*, and *Anaerosporobacter*, are indicative of normal glucose metabolism in pregnant women [62, 67, 90]. *Bifidobacterium* is known to produce metabolites that can modulate weight gain, improve insulin sensitivity and glucose tolerance, in addition to boosting the immune system [91–94]. In addition, levels of both *Bifidobacterium* and *Akkermansia* have been linked to gestational weight gain and high-fat dietary consumption before and during pregnancy, [56, 95], highlighting the impact of pre-pregnancy dietary intake in modifying the microbiota composition during pregnancy.

In first trimester overweight pregnant women, ketonuria, a marker of glucose metabolism, has been found to be associated with the relative abundance of specific bacteria including *Roseburia*, *Faecalibacterium*, and *Dialister* [96]. Previous research done with individuals with type 2 diabetes (T2D) has also shown decreased presence of *Roseburia intestinalis* and *Faecalibacterium prausnitzii* [68, 97]. Additionally, it has been discovered that, in first trimester overweight pregnant women, the genus *Collinsella* is positively correlated with insulin, c-peptide, and HOMA-IR (a marker of insulin resistance) [98]. On the other hand, the genus *Coprococcus* has been positively associated with the levels of GIP (gastric inhibitory peptide), a hormone that stimulates insulin secretion [98]. Additionally, the levels of hemoglobin A1C have been found to be positively correlated with the presence of *Bacteroides* and *Prevotella* [59] suggesting that changes in the composition of the gut microbiome may be utilized as clinical indicators to identify people with GDM who may require additional therapy. Research shows that commensal fungi have the potential to influence host metabolism directly [99]. Additionally, GDM has also been shown to impact the abundance of certain fungal species in the gut microbiota of pregnant women [100, 101], thus potentially modifying the interaction between bacteria and fungi. Recent studies have also found that specific bacterial and fungal patterns are associated with GDM development during early pregnancy and could help to identify women in higher risk of GDM development [101]. In regards to the gut microbiota of women suffering from GDM complex bacteria-fungi relationships were observed with concomitance of pathogenic fungi and pro-inflammatory bacteria, and also the co-occurrence of beneficial fungi and bacteria [102]. Evidence of dysregulated vaginal mycobiome and poor glycemic control was observed in one study with diabetic pregnant women demonstrating increased proportion of *Candida* spp. accompanied with decreased proportions of *Saccharomyces* spp. and uncultured fungi [103]. Further studies are needed to study the dynamic alteration and interaction of the fungal and bacterial components in GDM patients. Thus, in addition to the strategies that promote a beneficial bacterium the pregnancy mycobiota might also offer opportunity for intervention in order to improve inflammatory status and insulin resistance and the future health of the offspring.

### Role of microbial metabolites in GDM

SCFAs are produced by the gut bacteria via fermentation of dietary fibers [65]. SCFAs have been shown to reduce metabolic endotoxemia by upregulating the expression of tight junction proteins such as ZO-1 and occludin, which help to maintain the integrity of the intestinal barrier [104]. SCFAs have also been shown to enhance the function of regulatory T cells (Tregs) in the gut and provide strong anti-inflammatory functions essential for maintaining a healthy pregnancy [105, 106]. The lower abundance of the SCFA-producing genera of *Actinobacteria* in pregnant women with GDM shed the light on the importance of the bacterial metabolites in suppressing the pregnancy associated metabolic endotoxemia [107, 108].

Butyrate mediates anti-inflammatory effects in the intestinal mucosa by inhibiting the NF-κB transcription factor and activating CX3CR1 in macrophages [66, 105, 109, 110]. Butyrate also regulates TLR 4 gene expression by reducing the translocation of LPS and blocking LPS-stimulated dendritic cells, as well as enhancing the activity of Treg cells and inhibiting the immune response against the gut microbiota which is important for maintaining a healthy pregnancy [66, 105, 109, 110]. Moreover, butyrate is also reported to maintain the intestinal integrity by modulating the intestinal forkhead box protein P3 FOXP3 [111, 112].

Due to the beneficial effects of probiotics and SCFA in maintaining the immune-hemostasis in the gut, many clinical trials have been conducted in pregnant women with GDM, showing a significant role in controlling the glycemic and lipid levels in addition to the levels of the inflammatory markers within six weeks of administration [91, 92, 94, 113] (IRCT201704205623N108). Clinical trials have demonstrated that certain probiotic strains, such as *L. acidophilus* and *L. casei*, can improve the glycemic and lipid control while reducing some inflammatory markers within six weeks of administration [91, 92, 94, 113] (IRCT201704205623N108). Co-supplementation of probiotics with vitamin D or selenium has also been found to be effective in controlling glycemic levels in women with GDM [114, 115] (IRCT201706075623N119.IRCT20171010036697N1). These findings suggest that further clinical research is needed to identify potential microbiome-based therapies for different types of diabetes, including GDM. This suggests that prospective clinical studies are vital for the identification of potential novel microbiome-based therapeutic strategies for different types of diabetes including GDM. Moreover, previous studies had also described other gut bacteriome derived metabolites including hydroxybutyric acid, isobutyric acid, isovaleric acid, valeric acid, caproic acid in addition to the secondary bile acids, which showed a significantly higher levels in women with GDM, affecting glucose and lipid levels [116–119].These metabolites exhibited a positive correlation with OGTT blood glucose levels during the 24–28 weeks of gestation which was combined with increased abundance of proinflammatory bacterial species [116, 117]. Hydroxybutyric acid and Isovaleric acids are branched‐chain fatty acids (BCFAs), produced mainly during the fermentation of the branched‐chain amino acids (valine, leucine, and isoleucine) by some bacteria such as *Bacteroides* and *Clostridium* [120]. BCFAs have been shown to modulate the mucosal immunity of the host by influencing signaling pathways in gut epithelial cells [121]. Besides, they can also modulate the expression of genes that are involved in lipid synthesis and pro-inflammatory proteins production (mainly IL6) in obese individuals indicating that adipocytes inflammation and dyslipidemia can be mediated by a dysregulated BCFA profile [122]. Also, experimental studies on human and rats adipocytes explained the inhibitory role of BCFAs (mainly isobutyric acid) in the cAMP‐mediated lipolysis and insulin‐stimulated lipogenesis, affecting glucose and lipids metabolism in adipocytes [123]. Another study in diabetic rats reported an association between the inefficient glucose utilization and mobilization and the increased levels of 3-hydroxybutyric acid [124]. These studies highlighted the immunomodulatory and metabolic functions of BCFA in case of obesity, and since GDM is highly linked with the increased BMI suggesting a possible link.

In addition, other gut bacterial metabolites such as valine, leucine, isoleucine and tryptophan metabolites (kynurenic acid, hydroxy indoleacetic acid and Indolepropionic acid) exhibited also differential levels in women with GDM [125–127]. These metabolites are branched chain amino acids that showed to modulate the functions of T regulatory cells via mTORC1 dependent mechanism affecting different immunometabolic pathways necessary to maintain a healthy pregnancy [128].

Although, bile acids are synthesized by the host from cholesterol, these primary bile acids undergo metabolic transformation into secondary bile acids by the gut microbiota [129]. In regard to GDM, secondary bile acids including GUDCA, THDCA + TUDCA, and LCA‐3S exhibited a significantly higher serum levels, showing a positive correlation with glucose and triglyceride blood levels indicating a possible link with GDM [116, 125]. Bacterial bile acid metabolites such as isoallolithocholic acid (isoalloLCA) and 3-oxolithocholic acid (3-oxoLCA) were shown to modulate the balance between TH17 and Treg cells through the direct binding to retinoid-related orphan transcription factor along with enhancing the synthesis of the mitochondrial reactive oxygen species (mitoROS) in mice [130]. These metabolites are produced by a group of Bacteroides species including *Parabacteroides merdae*, *Bacteroides dorei* and *Bacteroides vulgatus* in addition to members of the Actinobacteria and Firmicutes phyla [131]. These bacteria convert the lithocholic acid (LCA) into 3-oxoLCA which in turn utilize the nuclear hormone receptor (NR4A1) to promote the differentiation of naive Tcells into regulatory T cells (Treg cells) [132], indicating the collaborative metabolic role of gut microbiota in maintaining the immune hemostasis. Interestingly, probiotic supplementation of *Lactobacillus rhamnosus* HN001 reduced the levels of the conjugated bile acids (which are the precursors of the secondary bile acids) in pregnant women with GDM, this can in turn enhance the glycemic control, showing greater effects among leaner and older women (age ≥ 35) [133].

A group of the important bacteriome‐derived metabolites is the B vitamins including pyridoxine (vitaminB6), folic acid (vitaminB9) and cobalamin (vitaminB12) which are de-novo synthesized by the gut microbiota [134, 135]. Previous studies in women with GDM indicated a higher level of folate during second or third trimester in which the higher maternal folate status combined with the B12 insufficiency can predict a higher risk of GDM [136–139]. In contrast, some studies revealed that Folic acid supplementation can protect early pregnant women from the risk of GDM, however, the exact dose or duration is yet to be identified [140]. Glycemic control by folic acid might be mediated via its anti-inflammatory and antioxidant effects [141–143], however, its exact mechanistic role in controlling hyperglycemia needs further investigations. Another important bacteriobiome‐derived metabolite is the Trimethylamine N‐oxide (TMAO) which is produce from molecules rich in choline [116, 144]. An exploratory metabolomic study highlighted a lower plasma TMAO concentrations in GDM subjects [145], additionally supplementation of TMAO to a high-fat diet mice showed to attenuate insulin signaling pathway in the liver, promoting inflammatory response in adipose tissue, and leading to insulin resistance and diabetes [146]. However, previous studies have also found a significantly positive correlation of the plasma levels of TMAO with the risk of GDM [144, 147, 148] thus in order to define the mechanistic role of TMAO in GDM further studies are required.

In addition, indole derivatives including indoleacetic acid (IAA) and indolepropionic acid (IPA) are tryptophan metabolites known to be regulated by gut microbiota (*mainly E.coli* and *lactobacilli*) [149, 150]. Among of these metabolites is the Indoleacetaldehyde which showed a significantly lower serum levels women with GDM [151]. Indoles were shown to modulate immunohomeostasis and inflammatory response in the gut affecting gut barrier permeability [150, 152] however, its exact role in GDM needs further investigations. These studies highlighted the significantly differentially expressed metabolites associated with the risk of GDM, providing insight into the pathogensis of GDM along with its predictive biomarkers.

### Impact of GDM on the development of the neonatal microbiome and infant health

Mother is the primary source of infant microbiome with the greatest exposure occurring during birth and post-partum period via both vertical and horizontal transmission [153, 154]. The first encounters with microbes may occur during pregnancy through intrauterine exposure which is followed by a significant microbial colonization of the newborn at birth [155]. The mother's vaginal, fecal, and skin microbiota are all transferred during this process of vertical transmission [7–9, 156]. Recently, Vatanem et al. have shown that a horizontal transfer of maternal microbiome mobile genetic elements takes place and contributes to shaping-up the offspring gut microbiomes [153]. Breastmilk is important for the development and maintenance of the infant microbiota after birth [11]. As the ‘‘typical’’ newborn microbiome develops, facultative anaerobes such as *Staphylococcus, Enterobacteriaceae* and *Streptococcus* are colonized first followed by obligate anaerobes such as *Bifidobacterium, Bacteroides,* and *Clostridium* [157]. These anaerobes play an important role in early immune maturation and host-gut cross-talk [158]. Maternal health status has a significant impact on the newborn’s gut microbiota, and early alteration of the microbiota has been linked to a number of inflammatory, dysmetabolic, allergy, and immune-mediated illnesses in later life [159].

Research has shown that infants born to GDM mothers are prone to develop obesity later in life [159]. Compared to infants born to non-GDM mothers, GDM infants demonstrate a significant reduction in the gut microbial diversity that could be indicative of dysbiosis [160]. Thus, in later life these newborns may be prone to developing gastrointestinal and metabolic diseases [160]. Furthermore, the microbial alterations observed in both mothers and neonates tend to be more similar in GDM effected pregnancies compared to controls, suggesting an intergenerational succession of microbial variations associated with GDM [14]. Table 2 summarizes the studies examining the gut microbiome of neonates/infants of GDM mothers compared to controls.

**Table 2:** Neonatal microbial signatures and GDM

Study/PMID	Location	Sample source	Subject details	Sequencing methodology	Main findings (Microbiota Signatures in GDM) compared to controls
[163]	China	Meconium sample	418 Mother-infant (147 with GDM and 271 normal pregnant women)	16S rRNA sequencing (V3 region)	Alpha diversity *Proteobacteria* *Firmicutes*
[164]	USA	2 weeks infant stool sample	46 Mother-infant (13 with GDM and 33 normal pregnant women)	16S rRNA sequencing (V1-V2 region)	*Lactobacillus* *Flavonifractor* *Erysipelotrichaceae* *Phascolarctobacterium*
[165]	China	Meconium samples	120 Mother-infants (60 with GDM and 60 normal pregnant women)	16S rRNA sequencing (V3 region)	Alpha and beta diversity *Enhydrobacter*, *Psychrobacter*, *Aerococcus*, *Faecalibacterium*, *Herbaspirillum*, *Pelomonas*, *Burkholderia-Caballeronia-Paraburkholderia* *Xanthobacter*, *Cytophaga*, *Serratia*, and *Actinomyces*
[166]	Brazil	Meconium samples	84 Obese mothers with infants (40 with GDM and 44 normal pregnant women)	16S rRNA gene sequencing (V4 region	No significant difference in diversity or taxonomy was observed
[14]	China	Meconium samples	83 Infant meconium samples from GDM and non GDM mothers	16S rRNA gene sequencing (V3-V4)	*Enterobacter, Hydrogenophilus, Weissella* *Corynebacterium, Escherichia, Enhydrobacter, Bacteroides, Brevundimonas, Paracoccus, Schiegelella, Enterococcus*

Decrease in relative abundance

Increase in relative abundance

Aberrant oral microbial composition in newborn was also linked to maternal GDM [161] and studies have revealed that the oral microbiome of infants born to mothere with GDM is enriched with specific bacteria, such as *Alistipes* and *Lactobacillus*, which are also prevalent in the gut microbiota of women with GDM and are linked to excessive weight gain during pregnancy [161]. While these variations typically vanish as the child gets older, some studies have found a relationship between maternal GDM and changes in the oral microbiota of neonates [162]. However, the above studies lack standardization of methodology, are not controlled for confounding variables and other factors that could affect the results. Thus, to better understand the relationship between GDM and the infant microbiome, carefully planned large scale cohorts and longitudinal studies are needed.

### Microbiota-based therapy

There are several ways in which the gut microbial composition and function can be modulated, with untargeted intervention such as diet intervention, syn-, pre-, pro-, and post-biotics, microbiota transfer, or by targeted interventions with certain drugs, phage therapy etc. Although mild changes in diet may only produce minimal changes in the gut microbiome [167], extreme changes in diet can lead to significant and reproducible shifts in microbial composition [168]. In some cases, personalized interventions may be necessary to improve blood glucose control and reduce the risk of metabolic disease [169]. For example, research has shown that women with GDM who followed dietary recommendations had reduced levels of *Bacteroides* and showed a better glycemic control [59].

Prebiotics are non-digestible substances that are selectively used by the gut microbes to produce health benefits for the host [170]. Fermentable fibers such as inulin, fructo-oligosaccharides, and galacto-oligosaccharides are commonly used as prebiotics [170]. Supplementation with inulin or inulin-propionate ester has been shown to improve insulin resistance in overweight or obese individuals [171]. In addition, inulin-type fructans have been shown to improve fasting blood glucose, glycosylated hemoglobin, and fasting insulin levels in prediabetic and type 2 diabetic patients [172]. While the benefits of prebiotics in metabolic disease have been demonstrated, further research is needed to fully understand their effects on the host [173].

Probiotics are live microorganisms that have a beneficial effect on the host when consumed in adequate amounts. *Bifidobacterium* and *Lactobacillus* are common types of probiotics that have been tested in numerous randomized placebo-controlled trials in T2D individuals, with results suggesting that probiotic intervention can improve glucose homeostasis [174–176]. The potential benefits of probiotics have also been explored in women with GDM, a meta-analysis of randomized controlled trials has shown significant reductions in homeostatic Model Assessment of Insulin Resistance (HOMA-IR), fasting blood sugar (FBS) [177] inflammatory markers such as CRP, TNF-α, and IL-6 [178] upon probiotic supplementation in women with GDM compared to placebo [94, 177, 178]. The beneficial effects of probiotics may sometimes require the co-administration of other microorganisms or substrates, leading to the creation of synbiotics. Synbiotic is defined as “a mixture comprising live microorganisms and substrate(s) selectively utilized by host microorganisms that confers a health benefit on the host” [177]. A synbiotic containing inulin, *Akkermansia muciniphila*, *Clostridium beijerinckii*, *Clostridium butyricum*, *Bifidobacterium infantis*, and *Anaerobutyricum hallii* improved glucose metabolism after a standard 3 h meal tolerance test in T2D individuals, and was associated with altered fecal gut microbial composition, specifically increased levels of *A. muciniphila* and *B. infantis* [179].

Postbiotics are inanimate microorganisms or their components that produce health benefits when consumed [180]. Supplementation with purified microbial metabolites, such as butyrate, alone or in combination with inulin, has been shown to have some beneficial effects on inflammatory status and cardiometabolic phenotypes in humans [181–183]. Infusions of SCFA mixtures have also been shown to increase fat oxidation, energy expenditure In overweight/obese men [184]. These findings suggest that microbiota-derived metabolites may improve host metabolism. More research is needed to investigate the efficacy and safety of SCFAs in preventing insulin resistance and inflammation associated with GDM. Postbiotics are pasteurized versions of probiotics or parts of microbial strains with health-promoting effects [185]. A pilot trial of pasteurized *A. muciniphila* and its membrane protein amuc_1100 showed positive effects on markers of human metabolism [186].

Several studies have investigated the use of fecal microbiota transplant (FMT) as a potential treatment for improving metabolic parameters. In one study, obese individuals with metabolic syndrome experienced an improvement in insulin sensitivity 6 weeks after receiving FMT with microbiota from lean donors [187]. In contrast, FMT with autologous microbiota did not improve insulin sensitivity. Other studies have also shown that FMT from lean donors can have a temporary positive effect on insulin sensitivity in obese individuals with metabolic syndrome, without causing significant changes in other metabolic parameters [188]. Another study in mice suggested that FMT from individuals with GDM could alter the recipient's metabolism [189]. However, further research is needed to fully understand the impact of FMT on metabolic diseases.

Bacteriophages, or phages, are viruses that can infect and kill specific pathogenic bacteria [190]. Phage therapy involves the use of these viruses to target and eliminate harmful bacteria, and it has been suggested that they may be used in the future to target dysbiotic parts of the microbiome in individuals with metabolic disorders [190]. CRISPR-Cas systems are the adaptive immune systems of bacteria and archaea that can be used to engineer probiotic strains of bacteria, eliminate target bacteria, or modify gene expression [191, 192]. However, the potential unintended consequences of such therapies need to be carefully considered.

**Fig. 3:**
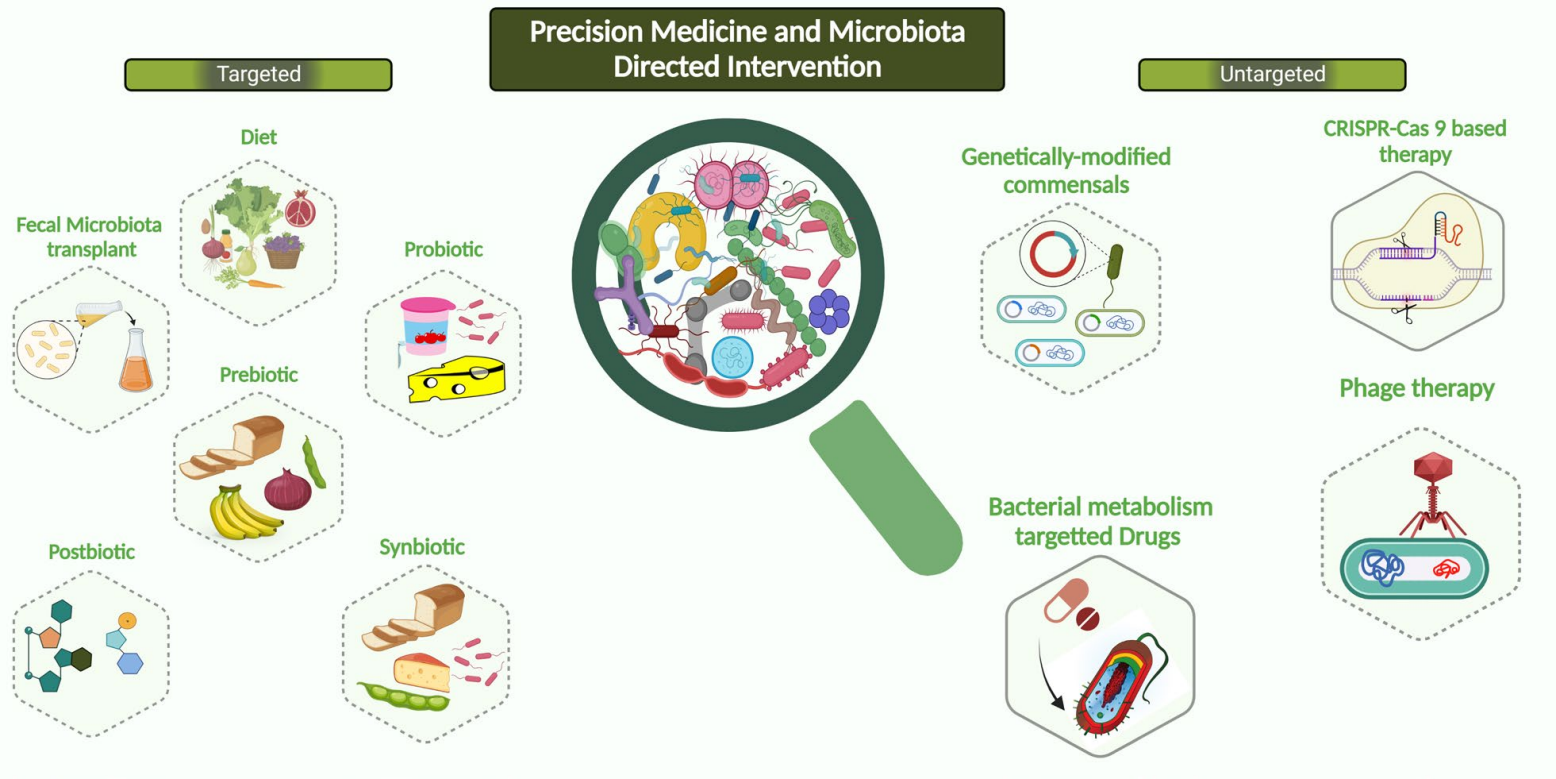
Gut microbiome and host-directed interventions for holistic control of metabolic health. Dietary interventions, prebiotics, probiotics, symbiotic, antibiotics, fecal microbiota transplantation, phage therapy, live biotherapeutics and microbiota-based medicines. Created with Biorender.com

Another approach to modifying the microbiome for the treatment of metabolic disorders is the use of drugs that target microbial metabolites or mimic their effects. For example, animal studies have shown that engineered *E. coli* that overexpress the satiety factor N-acylphosphatidylethanolamine can alleviate high-fat diet-induced obesity, insulin resistance, and hepatosteatosis in mice, and a genetically modified *L. gasseri* strain that can express and secrete glucagon-like peptide 1 (GLP-1) can increase insulin release and reduce hyperglycemia in rats with diabetes [193]. The use of microbiota-based therapies (summarized in Fig. 3) for the treatment of metabolic disorders is an active area of research, and although there are limitations to the current preclinical and clinical findings, focused research on these topics may revolutionize treatments in the future.

## Conclusion

Recent research has suggested that the microbiome plays an important role in the development and management of GDM. However, specific mechanisms by which the microbiome affects metabolism and how it may be modulated to improve health in women with GDM remain unknown. To better understand these relationships, it is important to design multi-omics studies aiming to dissect the pathophysiology of GDM in large pregnancy cohorts.

Additionally, well-powered, long-term randomized controlled trials with well-controlled groups of pregnant women with GDM will define the short and long-term dynamics of the microbiome and its interactions with other contributing factors. A complete understanding of the role of the microbiome in the development and management of GDM can pave the path to new ways to maintain metabolic health and prevent or treat GDM.

## Data Availability

Not applicable.
